# Atmospheric CO_2_ decline and the timing of CAM plant evolution

**DOI:** 10.1093/aob/mcad122

**Published:** 2023-08-29

**Authors:** Rowan F Sage, Ian S Gilman, J Andrew C Smith, Katia Silvera, Erika J Edwards

**Affiliations:** Department of Ecology and Evolutionary Biology, University of Toronto, 25 Willcocks Street, Toronto, ON, M5S 3B2, Canada; Department of Ecology and Evolutionary Biology, Yale University, New Haven, CT 06511, USA; Department of Biology, University of Oxford, South Parks Road, Oxford, OX1 3RB, UK; Department of Botany and Plant Sciences, University of California, Riverside, CA 92521, USA; Department of Ecology and Evolutionary Biology, Yale University, New Haven, CT 06511, USA

**Keywords:** CAM photosynthesis, drought adaptation, hydraulic luxury consumption, low atmospheric CO_2_, Miocene, Oligocene, photosynthetic evolution, succulence

## Abstract

**Background and Aims:**

CAM photosynthesis is hypothesized to have evolved in atmospheres of low CO_2_ concentration in recent geological time because of its ability to concentrate CO_2_ around Rubisco and boost water use efficiency relative to C_3_ photosynthesis. We assess this hypothesis by compiling estimates of when CAM clades arose using phylogenetic chronograms for 73 CAM clades. We further consider evidence of how atmospheric CO_2_ affects CAM relative to C_3_ photosynthesis.

**Results:**

Where CAM origins can be inferred, strong CAM is estimated to have appeared in the past 30 million years in 46 of 48 examined clades, after atmospheric CO_2_ had declined from high (near 800 ppm) to lower (<450 ppm) values. In turn, 21 of 25 clades containing CAM species (but where CAM origins are less certain) also arose in the past 30 million years. In these clades, CAM is probably younger than the clade origin. We found evidence for repeated weak CAM evolution during the higher CO_2_ conditions before 30 million years ago, and possible strong CAM origins in the Crassulaceae during the Cretaceous period prior to atmospheric CO_2_ decline. Most CAM-specific clades arose in the past 15 million years, in a similar pattern observed for origins of C_4_ clades.

**Conclusions:**

The evidence indicates strong CAM repeatedly evolved in reduced CO_2_ conditions of the past 30 million years. Weaker CAM can pre-date low CO_2_ and, in the Crassulaceae, strong CAM may also have arisen in water-limited microsites under relatively high CO_2_. Experimental evidence from extant CAM species demonstrates that elevated CO_2_ reduces the importance of nocturnal CO_2_ fixation by increasing the contribution of C_3_ photosynthesis to daily carbon gain. Thus, the advantage of strong CAM would be reduced in high CO_2_, such that its evolution appears less likely and restricted to more extreme environments than possible in low CO_2_.

## INTRODUCTION

Four photosynthetic carbon-concentrating mechanisms (CCMs) have been described in the Earth’s flora – C_4_ photosynthesis, dissolved inorganic carbon (DIC) concentration into pyrenoids or carboxysomes of algae and hornworts, C_2_ photosynthesis, and crassulacean acid metabolism (CAM photosynthesis). Each of these physiologies serves as an auxiliary to the ubiquitous C_3_ photosynthetic metabolism by transporting and concentrating CO_2_ around Rubisco, thereby suppressing photorespiration and enhancing carboxylation rate, which enables the enzyme to operate with greater efficiency (Sage and Stata, 2015). By functioning as a CCM, these mechanisms offset the reduction in atmospheric CO_2_ concentrations from >800 to < 500 ppm estimated to have occurred during the Oligocene epoch 23–34 million years ago (Ma) (Foster *et al*., 2017; Mills *et al*., 2019; Rae *et al*., 2021). Because of the advantages of CCMs at reduced CO_2_, it has been hypothesized that each originated when CO_2_ supply was restricted, either by low atmospheric values or high diffusion limitations around photosynthetic tissue (Ehleringer *et al.*, 1991, 1997; Raven *et al*., 2008; Arakaki *et al*., 2011; Sage *et al*., 2018). In the case of C_4_ photosynthesis, time-calibrated phylogenies and fossilized proxy records indicate most if not all C_4_ clades appeared after a CO_2_ decline in Earth’s atmosphere in the Oligocene (Christin *et al*., 2008, 2011; Vicentini *et al*., 2008; Sage, 2016). Models of C_4_ evolution also describe how low CO_2_ would cause high rates of photorespiration that in turn would promote intermediate steps in the evolution of C_4_ photosynthesis (Sage *et al*., 2012; Heckmann *et al*., 2013; Williams *et al*., 2013; Mallmann *et al*., 2014). Inorganic carbon concentration by algae is similarly hypothesized to have arisen during low CO_2_ episodes or in situations where photorespiration can be elevated, such as within dense algal mats in warm ponds or the interiors of stromatolites (Badger and Price, 2003; Griffiths *et al*., 2017; Raven *et al*., 2017). In the case of CAM photosynthesis, a few early phylogenetic studies addressing CAM origins and diversification support possible origins during low CO_2_ conditions of the post-Oligocene (Crayn *et al*., 2004 for bromeliads; Arakaki *et al*., 2011 for agaves and core cacti; see also Edwards and Ogburn, 2012; Heyduk, 2022). However, CAM evolution in non-flowering plant lineages, notably the lycophyte *Isoëtes*, the fern genus *Pyrrosia* and the gymnosperm *Welwitschia mirabilis*, has been hypothesized to be ancient (Cretaceous or earlier) given the antiquity of these lineages (Griffiths, 1989; Raven and Spicer, 1996; Winter and Smith, 1996; Keeley, 1998).

If low CO_2_ is an environmental enabler of CAM evolution, how strong is the evidence for its role, and how might it have promoted the evolutionary rise of CAM? This question can be addressed in greater depth today than a decade ago, due to advances in phylogenetic understanding in CAM-evolving lineages (e.g. Horn *et al*., 2014 for *Euphorbia*; Crayn *et al*., 2015 for bromeliads; and for orchids, Bone *et al*., 2015; Li *et al*., 2019; Gamisch *et al*., 2021). Large-scale carbon isotope surveys of CAM lineages have been forthcoming in the past two decades, often coupled with species-rich phylogenies that allow strong CAM to be mapped onto the phylogenies in greater detail (Silvera *et al*., 2005, 2010*a*, *b*; Bone *et al*., 2015; Li *et al*., 2019; Gamisch *et al*., 2021). Increasing numbers of time-calibrated phylogenies enable better estimates of when CAM may have originated within specific clades; these phylogenies often examine greater numbers of species using genomic data that can allow for greater precision in the dating of stem and crown nodes (Li *et al*., 2019; Gamisch *et al*., 2021; Wang *et al*., 2023). [Stem node refers to the phylogenetic node where a clade diverges from its sister clade, and crown node refers to the node that represents the most recent common ancestor of all extant species in the clade.]

In this review, we integrate the CAM phylogenetic, physiological and isotopic literature to develop a more comprehensive picture of the timing of CAM origins. In some cases, the resolution achieved in phylogenetic reconstruction and molecular dating allows for fairly precise estimates of CAM origins, at least in the case of species termed strong CAM, which are delineated by carbon isotope ratios (δ^13^C) less negative than −20 ‰ and in which the majority (>50 %) of daily C enters the plant at night via phosphoenolpyruvate (PEP) carboxylation (Winter and Holtum, 2002; Edwards, 2019). In numerous clades, where CAM is known to occur but its presence has not been systematically surveyed using δ^13^C or physiological assessments, we use node divergence dates to bracket the origin of a CAM clade, and by doing so establish a maximum age for CAM in that clade. We also address evidence for the origins of CAM in plants that typically acquire a majority of their carbon via C_3_ photosynthesis, and therefore exhibit δ^13^C values more negative than −20 ‰ that overlap with C_3_ plants. These weaker CAM plants have been termed ‘C_3_+CAM’ (Edwards, 2019). Here, we use this term to encompass all forms of weak, low-level CAM activity (whether constitutive or facultative), including the CAM idling and CAM cycling functional types (Winter, 2019). Weak CAM functional types presumably pre-date strong CAM functional types during CAM evolution, possibly as far back as the higher CO_2_ epochs before the Oligocene (Griffiths, 1989; Arakaki *et al*., 2011; Edwards, 2019). We next consider the radiation of CAM species after CAM acquisition. The CAM flora is estimated to comprise some 17 000–20 000 species, or nearly 7 % of all vascular plants (Gilman *et al*., 2023). This diversity evolved after CAM origins, and often appears to have been rapid as clades exploited the novel CAM physiology and changing environments.

We next address how low CO_2_ may have enabled CAM evolution, through consideration of low and high CO_2_ effects on C_3_ and CAM photosynthesis in extant species. We hypothesize that low CO_2_ may have enabled C_3_ ancestors to evolve traits such as leaf succulence that predisposed subsequent CAM evolution. We acknowledge that numerous other environmental factors such as heat, drought, elevated salinity and high evapotranspiration probably also played important roles in CAM evolution, possibly in tandem with low CO_2_. However, because our main objective is to evaluate whether reduced CO_2_ could have been a contributing factor for CAM evolution, we discuss these other potential drivers in less detail. A comprehensive treatment that fully addresses the multiple factors contributing to CAM origins is clearly needed, but this will probably require a robust model of how CAM evolved, clearer delineation of the early steps in CAM evolution and a more comprehensive phenotyping of CAM trait acquisition across multiple phylogenies (Edwards, 2023). We close with some thoughts on how the CAM flora may fare in a world of elevated atmospheric CO_2_, when the relative importance of these hypothetical drivers for CAM evolution and diversification may be strongly modified.

## ATMOSPHERIC CO_2_ AND THE ORIGIN OF CAM – THE EVIDENCE

**Fig. 1.**
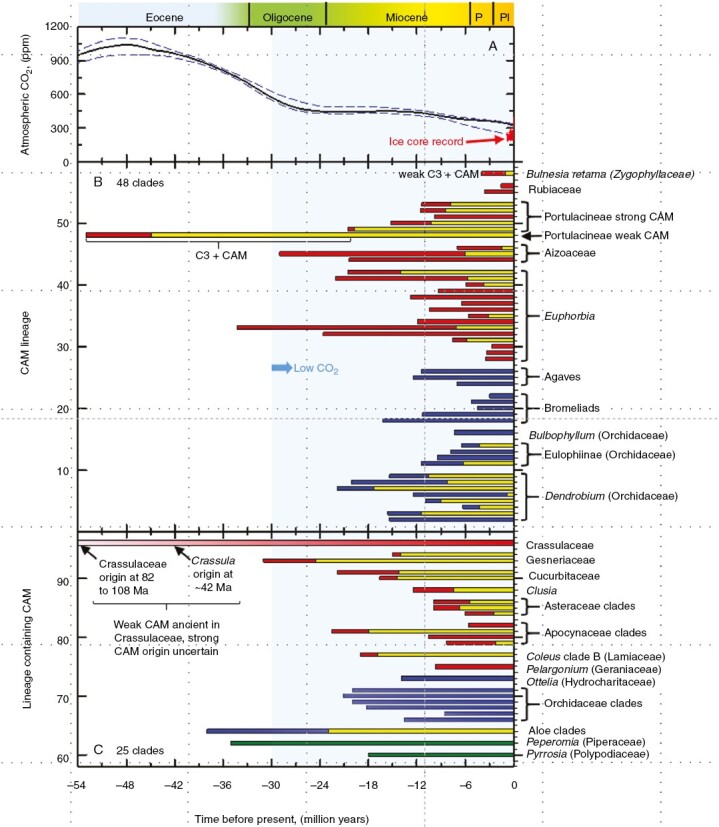
(A) Proxy-based estimates of atmospheric CO_2_ for the past 54 million years, from fig. 4b in Foster *et al.* (2017). Solid lines show the 50 % quantile estimates, and dashed lines show the 2.5 and 97.5 % quantile estimates. (B) Age estimates for CAM acquisition in lineages containing CAM species. A full bar length represents stem divergence dates from the source chronograms or stem dates stated within the referenced source. Blue-toned bars indicate stem ages for monocot lineages, and red-toned bars indicate stem dates for eudicot lineages. Where crown dates are available, they are also shown in all cases as yellow bars within the longer stem-date bars. (C) Age estimates for clades that contain C_3_ and CAM taxa, where the time of CAM acquisition cannot be inferred. The dates thus estimate the earliest possible origin of CAM in the clade, not necessarily when CAM did arise. In most clades, CAM origins would occur later than the bars indicate. Stem node dates of monocots are indicated with blue-toned bars, and for eudicots, as red-toned bars. The green-toned bars indicate the stem node dates for *Pyrrosia* ferns and *Peperomia,* a genus in the early angiosperm family Piperaceae. See Supplementary Data Appendix S1 for methods and literature sources. Abbreviations: P, Pliocene; PL, Pleistocene. Legend scales and tick marks in B and C correspond to specific clades as follows. In B, tick marks 2–9 correspond to *Dendrobium* CAM clades 1–8 (Li *et al*., 2019), while ticks 11–14 indicate Eulophiinae CAM clades 1–4 from Bone *et al.* (2015). The remaining clades in B are as follows (tick numbers in parentheses): *Bulbophyllum* (16); the bromeliad clades Hechtioideae (18), *Dyckia* (19), *Puya*/core bromeliads (20), core *Tillandsia* (21) and *Tillandsia utriculata* (22); *Agave* clades *Hesperaloe* (24), *Yucca* crown (25) and *Agave* (26); *Euphorbia* clades *E. mauritanica* (28), *E. schimperi* (29), *E. laterifolia* (30), section Anthacanthae stem 8 (31), *E. platyclada* (32) and section Articulofruticosae (33); *E. ceroderma* (34), subsection Tirucalli (35); *E. weberbaueri* (36); *E. lomelii* (37); *E. sipolisii* (38); *E. alluaudii* (39), section Goniostema (40), section Monadenium (41) and section Euphorbia (42); Aizoaceae clades *Tetragonia* (44), Mesembryanthemoideae (45) and core Ruschioideae (46); Portulacineae (48); core Cactoideae (49), *Tephrocactus*–*Opuntia* (50), *Grusonia* (51), Didiereaceae (52) and *Anacampseros* (53); Rubiaceae clades *Myrmecodia* (55) and *Squamellaria* (56); and *Bulnesia retama* in Zygophyllaceae (58). In C, the order of lineages are (tick position in parentheses): *Pyrrosia* (60); *Peperomia* (62); aloids (64); Orchidaceae clades Maxillariinae (66), Stanhopeinae (67), Oncidiinae (68), Laeliinae (69), Vandeae (70) and Pleurothallidinae (71); *Ottelia* (73); *Pelargonium* (75); *Coleus* clade B in the Lamiaceae (77); Apocynaceae clades *Ceropegia* (79), *Cynanchum* (80), *Hoya–Dischidia* (81) and *Pachypodium* (82); Asteraceae clades *Caputia* (84), *Crassothonna* (85) and the Gynurids (86); and *Clusia* (88). The Cucurbitaceae clades are *Seyrigia* (90) and *Xerosicyos* (91); and Gesneriaceae are clades Ramondinae (93) and *Codonanthe*–*Codonanthiopsis*–*Nematanthus* (94). The family Crassulaceae (96) is shown to represent probable ancient origins of C_3_ + CAM that extend to near the origin of the family, estimated to occur between 82 Ma (crown node age) and 108 Ma (stem node).

A role for reduced atmospheric CO_2_ in promoting CCM evolution was first proposed by Ehleringer *et al.* (1991) for C_4_ origins and hypothesized for CAM origins by Raven and Spicer (1996), Keeley and Rundel (2003), Crayn *et al.* (2004) and Arakaki *et al.* (2011). Atmospheric CO_2_ is modelled to have declined from elevated (>800 ppm) to near current values (300–400 ppm) during the Oligocene (34–23 Ma; Fig. 1A; Tipple and Pagani, 2007). Consistently, nearly all C_4_ origins are predicted by phylogenetic dating to have followed the late Oligocene decline in atmospheric CO_2_, and a surge in C_4_ evolution is evident in the late Miocene, 5–10 Ma (Fig. 1A; Christin *et al*., 2008, 2011; Vicentini *et al*., 2008; Sage *et al*., 2018). The Oligocene and later Miocene are also recognized as times when climates deteriorated, with many lower latitude regions drying, causing forests to retreat and a commensurate expansion in savanna grasslands and semi-arid, open landscapes (Tipple and Pagani, 2007; Osborne and Freckleton, 2009; Edwards *et al*., 2010). In response to this low- to mid-latitude aridification during the Oligocene and then later in the Miocene, xerophytic traits appeared in a wide range of plant taxa, and from these clades, many of the existing C_4_ and CAM lineages evolved, indicating that adaptations to drought in ancestral C_3_ populations also enabled CCM evolution and diversification (Sage, 2001, 2002; Arakaki *et al*., 2011; Christin *et al*., 2013; Griffiths *et al*., 2013; Sage *et al*., 2018). Because CCM taxa largely appear in regions with warm and dry climates, or in locally saline conditions, it is generally recognized that discussions of low CO_2_ influences on CAM and C_4_ evolution should be considered in the context of environmental stressors such as heat, drought, salinity, fire disturbance, or soil-less environments such as rock outcrops and epiphytic niches (Raven and Spicer, 1996; Sage, 2001; Crayn *et al*., 2004; Tipple and Pagani, 2007; Edwards and Smith, 2010; Edwards *et al*., 2010).

### The timing of CAM origins

Does a similar timing exist for CAM origins as observed for C_4_ origins? To address this, we compiled age estimates for 48 strong CAM clades delineated by δ^13^C and one weaker C_3_ + CAM clade identified using gas exchange and titratable acidity (Fig. 1B). We also show divergence time for clades known to contain CAM species, but in which specific CAM nodes are difficult to discern given current levels of phylogenetic resolution (Fig. 1C). In the latter case, the CAM origin(s) within a clade are not known precisely, but would have corresponded to or post-dated the clade origin. Sources and assumptions used in preparing Fig. 1 are presented in online Supplementary Data Appendix S1.

In the data presented in Fig. 1B, strong CAM is emphasized because it can be readily delineated with δ^13^C values less negative than −20 ‰ obtained from herbarium specimens and living plants (Winter and Holtum, 2002; Winter *et al*., 2015; Winter, 2019; Messerschmid *et al*., 2021). Strong CAM plants also exhibit high degrees of succulence in photosynthetic tissues, reflecting the contribution of succulence to CAM function (Kluge and Ting, 1978; Nelson and Sage, 2008; Edwards, 2019; Luján *et al*., 2022; Leverett *et al*., 2023); however, succulence also occurs in C_3_, C_4_ and weak CAM plants, so by itself is an uncertain indicator of strong CAM (Griffiths and Males, 2017; Borland *et al*., 2018; Leverett *et al*., 2023). We thus had to treat claims of CAM based on succulence alone with caution. The origins of C_3_ + CAM physiology are more difficult to delineate because of the overlap between more negative CAM values and typical C_3_ values of δ^13^C, so they require physiological assessments of CAM activity, notably nocturnal acid accumulation, diel patterns of CO_2_ fixation and/or malate accumulation (Winter, 2019; Winter and Smith, 2022). Because physiological assessments require living plant material, and CAM activity in facultative species may only be revealed when plants are subject to the appropriate degree of water deficit, surveys for C_3_ + CAM function are often incomplete for a given clade; in such cases, mapping C_3_ + CAM onto a phylogenetic tree may not clearly identify where CAM originates within the phylogeny. Hence, the inferred age of the entire clade containing C_3_ + CAM species is often the only possibility for estimating the earliest possible CAM origins, as presented for numerous clades in Fig. 1C.

Altogether, origin estimates for 73 CAM clades or lineages containing CAM are presented in Fig. 1, whilst recognizing that the true number of independent origins could be greater or less depending on alternative interpretations of certain phylogenies (Gilman *et al*., 2023). In Fig. 1B, 46 lineages of strong CAM are estimated to have originated during the past 30 Ma when atmospheric CO_2_ concentration had declined to current values of 424 ppm or less (June 2023 CO_2_ values, www.co2earth). Over 90 % of the CAM lineages in Fig. 1B are estimated to be younger than 25 Ma, which corresponds to a period after Oligocene CO_2_ concentration is modelled to have declined to below 400 ppm (Fig. 1A). Estimates for two CAM clades – the *Euphorbia* clade Articulofruticosae and subfamily Mesembryanthemoideae of Aizoaceae – extend back to the Oligocene when atmospheric CO_2_ was in decline. One large clade – the Portulacineae – appears to have a relatively ancient origin of weak C_3_ + CAM extending to near 50 Ma, when atmospheric CO_2_ concentrations were high, being two to three times those of Miocene to present-day values. The Portulacineae is a large suborder of eight mostly succulent families in the Caryophyllales containing multiple clades of CAM species, including the Cactaceae, Didiereaceae and the C_4_ + CAM species in the Portulacaceae (Nyffeler and Eggli, 2010; Wang *et al*., 2019; Gilman *et al*., 2023). The appearance of strong CAM in some individual Portulacineae clades such as *Anacampseros*, the Cactaceae and the Didiereaceae corresponds to the Miocene, when atmospheric CO_2_ was reduced (Fig. 1B). Most of the speciose CAM clades, notably *Euphorbia* and Aizoaceae of the Palaeotropics, bromeliads and agaves of the Neotropics, and both Palaeo- and Neotropical orchids form CAM clades in post-Oligocene time. Numerous small clades of CAM species, such as two clades in Rubiaceae subtribe Hydnophytinae, are relatively young, evolving CAM in the Pliocene or later (Fig. 1B). A newly discovered C_3_ + CAM xerophyte in the Zygophyllaceae with photosynthetic stems, *Bulnesia retama* (Mok *et al*., 2023), is estimated to have diverged from non-CAM sisters some 1–4 Ma (Böhnert *et al*., 2020).

We were also able to use age estimates for 25 clades containing CAM that have sufficient precision to allow us to conclude CAM evolved after cladogenesis (Fig. 1C). The genus *Peperomia* (Piperaceae) originated in the high CO_2_ conditions of the Eocene at 40–50 Ma (Naumann *et al*., 2013; Massoni *et al*., 2015), but phylogenetic, isotopic and physiological details are currently too sparse to indicate when the CAM clades within this large genus (>1400 spp.) appeared. As this information is acquired, CAM in *Peperomia* might turn out to occur in multiple, disparate clades of relatively young age that date to epochs of reduced CO_2_, or it could be more widespread, indicating fewer, more ancient origins extending to the Eocene. Six large orchid clades containing CAM species are estimated to have Miocene origins, allowing us to conclude the CAM origins are Miocene or younger in these lineages (Fig. 1C). As an example of how much younger a specific CAM origin may be relative to a clade age, we consider the case of the Dendrobiinae, a tropical clade of orchids estimated to have a stem age of 32 Ma (Gustaffson *et al*., 2010; Givnish *et al*., 2015). A detailed study of CAM occurrence within *Dendrobium* indicates that CAM clades within the Dendrobiinae subtribe emerged during the late to early Miocene, a period characterized by reduced atmospheric CO_2_ levels (Li *et al*., 2019). As a result, these CAM clades are hypothesized to be considerably younger than the estimated node age for this subtribe (Fig. 1B; Li *et al*., 2019). Similarly, the orchid clade Pleurothallidinae stem age is 14.2 Ma (Givnish *et al*., 2015), yet three CAM clades diverged within this subtribe in the undated phylogeny of Silvera *et al.* (2009), indicating younger ages for CAM. In the Vandeae orchids, CAM is present in *Campylocentrum* (Silvera *et al*., 2009), whose crown node is subtended by a long branch to the stem node, indicating a much younger age than the Vandeae age shown in Fig. 1C (Givnish *et al*., 2015). We envisage this pattern of younger ages for CAM nodes than the clade age will be commonly observed in other clades once CAM phenotyping is complete (i.e. testing for CAM activity in living material) and the precise origins of CAM can be mapped onto the phylogenies. For now, we emphasize that the estimates in Fig. 1 are based on surveys involving a minority of species in what are often species-rich clades. Changes to these estimates are likely as taxonomic and physiological sampling improve and allow for more precise dating and species placement with the respective phylogenies.

The large monocot clade of CAM species in Asphodelaceae subfamily Alooideae includes the notable CAM genus *Aloe* (>500 spp.), plus 11 other mainly succulent CAM genera, including a number recently segregated from *Aloe* (Grace *et al*., 2015). Members of the other subfamily Asphodeloideae generally lack pronounced succulence, but weak CAM activity has been reported in the genus *Bulbine*, which has been resolved in a sister-group relationship to the alooids (Grace *et al*., 2015; Gilman *et al*., 2023). The crown node date for the separation of the Alooideae and Asphodeloideae is near 23 Ma, when CO_2_ was reduced (Fig. 1C). However, the stem age for the family is considerably older, near 38 Ma, when CO_2_ was elevated. The genus *Aloe* and related alooids represent a large clade of CAM species that has yet to be fully characterized, but focused work on the early diverging alooids and their relationship to the asphodeloids could clarify the timing and nature of the CAM origin in this major CAM clade.

Figure 1C also shows age estimates for three clades that contain weaker C_3_ + CAM species. *Ottelia* from the Hydrocharitaceae contains aquatic CAM species that are probably younger than its 13.9 Ma node age (Li *et al*., 2020), while *Pelargonium* in the Geraniaceae is estimated to have diverged 9.7 Ma (van der Kerke, 2019). The genus *Clusia* (Clusiaceae) exhibits a wide range of CAM phenotypes (Lüttge, 2007*a*; Luján *et al*., 2023), from weaker C_3_ + CAM to strong CAM species, and is estimated to have stem and crown dates of 12.5 and 7.5 Ma, respectively, so the multiple origins of strong CAM in this well-studied clade correspond to the late Miocene or later (Luján *et al*., 2022). Strong CAM probably originated in *Clusia* three to five times, all probably in the past 5 million years from C_3_ + CAM ancestors that branch near the base of the *Clusia* phylogeny (Ruhfel *et al*., 2016; Luján *et al*., 2022). Because of the extensive physiological and ecological research already on *Clusia* and its high number of diverse CAM character states (Lüttge, 2007*a*; Borland *et al*., 2018; Leverett *et al*., 2023; Luján *et al*., 2023), the genus could become an excellent model for CAM evolution with further phylogenetic resolution.

Most clades containing CAM species have received some level of phylogenetic investigation, but limited physiological assessments prevent a clear picture of CAM origins. In these cases, estimates of clade origins allow us to establish a putative oldest boundary on CAM age. In the Apocynaceae, CAM is estimated to have arisen four times in distinct clades. The CAM *Hoya*–*Dischidia* clade is sister to the non-CAM genus *Oreosparte*, implying a CAM origin at or later than this split, which is dated to the early Miocene (Fig. 1C; Liede-Schumann *et al*., 2022). *Ceropegia* is part of a large clade containing the stem-succulent stapeliads and its origin can be estimated at 2–8 Ma (Fig. 1C; Bruyns *et al*., 2015). Similarly, the clade containing the CAM genera *Cynanchum* and *Pachypodium* are each nested in presumably non-CAM clades with divergence estimates at 10.6 and 5.6 Ma, respectively, although the CAM assignation for *Pachypodium* currently rests upon a single species, reported to be weak C_3_ + CAM (von Willert *et al*., 1980, 1992). In the Asteraceae, each of the three clades in which CAM has been documented (*Caputia*, *Crassothonna* and the Gynurids) are nested within distinct non-CAM clades with divergence dates estimated at 5–10 Ma (Fig. 1C; Pelser *et al*., 2010). The Gynurid clade is one of the prominent southern African and Madagascar CAM clades, containing species in *Senecio*, *Curio* and *Kleinia* (Gilman *et al*., 2023). In the Cucurbitaceae, CAM occurs in two distantly related genera nested in C_3_ clades – *Seyrigia* and *Xerosicyos*, both endemic to Madagascar – which are dated to 14–22 Ma (Guo *et al*., 2020). The two genera are relatively species-poor (six species each), and thus may be relatively simple and tractable systems in which to study CAM evolution. In the Gesneriaceae (African violet family), two CAM lineages are identified, one in subtribe Ramondinae (the *Haberlea*–*Ramonda* clade), and the other nested within subtribe Columneinae (the *Codonanthe*–*Codonanthopsis*–*Nematanthus* clade). Petrova *et al.* (2015) date the Ramondinae spilt from a non-CAM sister clade at a crown age of 24.5 Ma, with a stem node of 30.5 Ma, whereas in the dating analysis of Roalson and Roberts (2016) the equivalent stem node is at 41.5 Ma. The other CAM-inclusive lineage, the *Codonanthe*–*Codonanthopsis*–*Nematanthus* clade, is dated by Roalson and Roberts (2016) to a stem node at 14–15 Ma, but the only CAM taxon so far identified within this clade, *Codonanthopsis crassifolia* (formerly *Codonanthe crassifolia*: Guralnick *et al*., 1986), appears younger, diverging 3–6 Ma and showing weak C_3_ + CAM. Relative to their large size (~3800 spp.), the Gesneriaceae have so far been severely undersampled for CAM activity and would merit further study, especially as the family contains many tropical epiphytes and climbers.

A more prominent family of CAM is the Crassulaceae. In addition to providing the name for the photosynthetic pathway, the Crassulaceae has many important clades of strong and weak CAM, in genera such as *Aeonium*, *Cotyledon*, *Crassula*, *Kalanchoë*, *Sedum* and *Sempervivum* (Smith and Winter, 1996; Gilman *et al*., 2023). A wide range of CAM character states are reported in most major subclades of the family, based on gas exchange, nocturnal acidity accumulation and carbon isotope ratios; succulence is also widespread (Kluge and Ting, 1978; Lösch, 1984; Pilon-Smits *et al*., 1996; Smith and Winter, 1996; Messerschmid *et al*., 2020). When mapped onto the Crassulaceae phylogeny, strong CAM is evident in clades with nodes deep within the Crassulaceae, supporting a possibility that CAM is ancient, evolving in some lineages during episodes of high atmospheric CO_2_. Recent phylogenetic investigations, however, estimate widely varying divergence times for the family and major subclades. Bruyns *et al.* (2019) infer a Crassulaceae crown age of 44.9–65.0 Ma, while Messerschmid *et al.* (2020) estimate the Crassulaceae diverged 81.7 Ma (crown node) to 107.5 Ma (stem node). While these dates vary substantially, both suggest a pre-Oligocene origin of at least weak CAM in Crassulaceae, when atmospheric CO_2_ was 750–1000 ppm. In addition to the broad age estimates, pinpointing the origins of CAM in the Crassulaceae becomes particularly difficult due to uncertainty over the phylogenetic distribution of the strong and weak CAM clades. This uncertainty is represented in Fig. 1C by a bar with faded shading that extends beyond the Fig. 1C panel. For example, *Crassula* probably pre-dates the Oligocene based on crown nodes of 36 Ma (Lu *et al*., 2022), 40 Ma (Messerschmid *et al*., 2020) and 46 Ma (Bruyns *et al*., 2019). Strong CAM has been demonstrated in a handful of *Crassula* species of the highly succulent clades B and C (Kluge and Ting, 1978; Bruyns *et al*., 2019), leading Bruyns *et al*. (2019) to hypothesize that CAM (presumably strong CAM) arose in these clades with the acquisition of pronounced succulence. If true, age estimates for clades B and C of 4–16 Ma would indicate a strong CAM origin in the late Miocene to early Pliocene (Bruyns *et al*., 2019; Lu *et al*., 2022). Even the older dates of Messerschmid *et al.* (2020) suggest that most extant Crassulaceae subclades containing strong CAM diversified after the early Oligocene (e.g. the strongly succulent subfamily Kalanchoideae has a crown age of 32.9 Ma). Moreover, in *Aeonium*, Messerschmid *et al.* (2023) using phylogenomic-scale data estimated a crown age of just 1.7‒8.1 Ma, roughly 40 million years younger than predicted by Messerschmid *et al.* (2020) using ITS and three plastid markers. While broader isotopic surveys and phylogenetic taxon sampling are clearly needed to firmly delineate the strong CAM clades in Crassulaceae, the currently known diversity of CAM and pronounced succulence in each subclade suggests that, even if the earliest CAM taxa may have originated in the Cretaceous, many lineages were primed for rapid evolution of strong CAM in more recent epochs.

Many families with CAM species were not considered in Fig. 1C due to uncertainties in clade age and CAM occurrence within the phylogeny, which made estimations of CAM origin difficult. The Vitaceae provide a case in point. Here, the speciose yet distinct genera *Cissus* (~290 spp.) and *Cyphostemma* (~240 spp.) contain at least a few CAM species (Virzo De Santo *et al*., 1983; Virzo De Santo and Bartoli, 1996). In *Cissus*, six CAM species occur in three disparate clades, with four species including *C. quadrangularis* occurring in a succulent African clade of Miocene age (Liu *et al*., 2013). A survey of online images of *Cissus* by the authors suggest typical C_3_ morphology is common in the genus, yet C_3_ determinations are not reported; hence, where and when CAM might have originated in the phylogeny is unknown, but is probably later than the estimated genus age of 62 Ma (crown) to 72 Ma (stem) (Liu *et al*., 2013). Similarly, in *Cyphostemma*, CAM is documented in three species from southern Africa, all of which occur in a clade of caudiciform stem succulents, suggesting this entire succulent clade is CAM. Hearn *et al.* (2018) date this clade to 16.3 Ma, much younger than their estimated age for *Cyphostemma* of 44 Ma (crown) to 63 Ma (stem). Again, because the taxonomic distribution of C_3_ species is unknown, it is not possible to clearly identify where and when the CAM lineages have originated in *Cyphostemma*. The Vitaceae example highlights the need to report not just positive CAM identifications, but also C_3_ identifications in surveys of CAM photosynthesis.

With respect to when CAM evolved in non-flowering plant lineages, we consider possibilities for the lycophyte *Isoëtes*, the fern genus *Pyrrosia*, and the gymnosperms *Welwitschia* and *Dioon* where phylogenetics and physiology allow for limited inferences. CAM is believed to be ancestral in *Isoëtes*, which is estimated to have a crown age of 45‒65 Ma and a stem potentially extending to ~370 Ma (Wood *et al*., 2020). However, the evolution of CAM in *Isoëtes* is probably an adaptation to the low diffusion potential of CO_2_ in aqueous environments, rather than in response to declining atmospheric CO_2_, as evidenced by a phenotypic transition to exclusively C_3_ photosynthesis when growing terrestrially (Keeley, 1998). In the epiphytic fern genus *Pyrrosia*, C_3_ + CAM has been reported in a clade of plants with deeply sunken stomata that is specific to Southeast Asia, Indonesia and northeastern Australia, with an estimated mean divergence time of 18 Ma (Fig. 1C; Wei *et al*., 2017). CAM has not been reported for other *Pyrrosia* clades from Africa and Asia, suggesting CAM originated in the clade with sunken stomata (Wei *et al*., 2017). The sister genus to *Pyrrosia*, *Platycerium*, is reported to contain very weak CAM (Holtum and Winter, 1999), but the C_3_*Pyrrosia costata* clade is sister to both the *Pyrrosia* clade with CAM and *Platycerium*, indicating distinct CAM origins (Wei *et al*., 2017). Weak C_3_ + CAM has been reported in the monotypic gnetophyte *Welwitschia mirabilis* (reviewed in von Willert *et al*., 2005). The family Welwitschiaceae is considered ancient, dated by fossils to at least the lower Cretaceous; however, these fossils lack xeromorphic characters and some even appear aquatic (Jürgens *et al*., 2021). These characters suggest CAM was unlikely in ancestral *Welwitschia*, and it may have arisen more recently as the ancestors of the surviving *W. mirabilis* plants became adapted to arid African landscapes. Similarly in the cycads, another ancient lineage of gymnosperms, diversification into more arid habitats appears to have occurred in the Miocene, although weak C_3_ + CAM activity (CAM cycling) has so far been reported only in the single taxon *Dioon edule* (Vovides *et al*., 2002; Gutiérrez-Ortega *et al*., 2018).

**Fig. 2.**
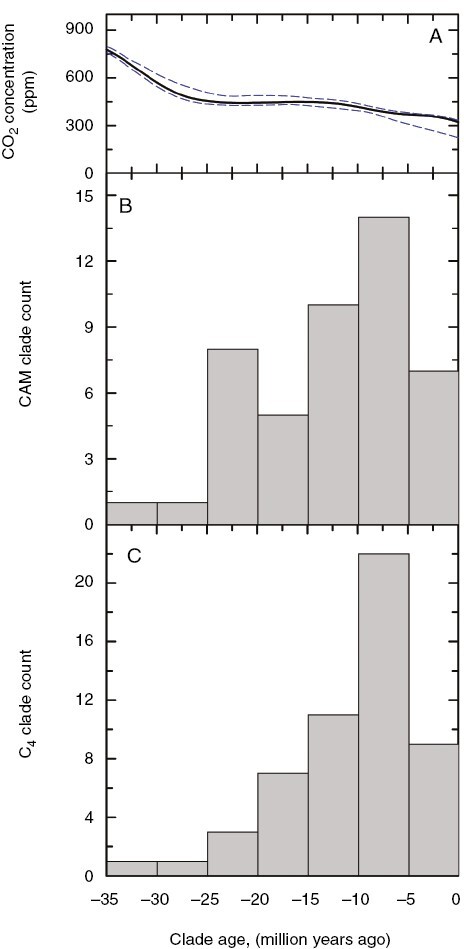
Frequency diagram showing when the estimated strong CAM and C_4_ lineages originated over the past 35 million years. (A) CO_2_ history shown in Fig. 1A. (B) Origin estimates of strong CAM clades from Fig. 1B, using divergence dates (usually stem node estimates) given in the cited papers. C_4_ clade values are from Sage (2016).

Using the age estimates in Fig. 1B, we present a frequency diagram of strong CAM origins and compare it with origin estimates of C_4_ clades presented by Sage (2016; Fig. 2). For both CAM and C_4_, the majority of lineages are estimated to have arisen in the Miocene or later, with a peak occurring in the late Miocene at 5–10 Ma. The late Miocene also corresponds to the interval when C_4_-dominated grass biomes expanded across low to mid-latitudes (Cerling *et al*., 1997; Edwards *et al*., 2010). This synchronous spike in C_4_ origins and expansion of C_4_ grasslands during the Miocene is viewed as being enabled by the establishment of low CO_2_ conditions, but other factors such as increasing aridity, seasonality, fire frequency and the radiation of large herbivore guilds may have played a triggering role (Osborne and Freckleton, 2009; Edwards *et al*., 2010; Charles-Dominique *et al*., 2016). A recent review of the atmospheric CO_2_ literature also estimates CO_2_ continued to drift downward from the mid-Miocene Climatic Optimum (~16.9‒14.7 Ma) during the late Miocene, from near 500 to 400 ppm (Rae *et al*., 2021; Steinhorsdottir *et al*., 2021). A combination of stresses, notably low CO_2_, heat, salinity and drought, are now thought to have promoted the initial origins of C_4_ photosynthesis, because together they cause high rates of photorespiration in C_3_ plants, leading to more glycine production and the release of photorespiratory CO_2_ and ammonia at the glycine decarboxylation step (Sage *et al*., 2018). Drought and salinity reduce stomatal conductance, which acts in concert with low atmospheric CO_2_ to further reduce chloroplast CO_2_ concentration, further enhancing photorespiration (Sage, 2013). To compensate for high rates of photorespiration, certain plants evolved mechanisms to trap and refix photorespired CO_2_, one of which is C_2_ photosynthesis wherein photorespiratory glycine is shuttled from the mesophyll to bundle sheath cells, at which point it is decarboxylated to yield CO_2_ and ammonia (Monson and Rawsthorne, 2000; Sage *et al*., 2012). The C_4_ biochemical cycle is hypothesized to have initially appeared to assist in the recovery of photorespired ammonia liberated in the bundle sheath cells by glycine decarboxylation, after which it was upregulated to form a strong CCM (Mallmann *et al*., 2014; Adachi *et al*., 2023). This incipient C_4_ metabolism became an exaptation for C_4_ pathway evolution, because its original role was to facilitate the function of the C_2_ pathway, but in doing so it enabled the subsequent rise of CO_2_ concentration into the bundle sheath. In considering CAM evolution, and how declining CO_2_ may have influenced it, it will also be worth considering how aspects of the C_3_ physiology became exaptations for subsequent CAM evolution (Edwards, 2023). For example, succulence of chlorenchyma cells to enhance hydraulic capacitance and stomatal conductance in C_3_ ancestors could have been one exaptation that facilitated nocturnal malate accumulation (Griffiths, 1989; Edwards, 2019).

### The timing of CAM diversification

If low CO_2_ favoured CAM evolution, then this would inevitably also have promoted species diversification within CAM lineages as they exploited the new physiology and radiated into a wide range of environments and growth forms (Crayn *et al*., 2004; Arakaki *et al*., 2011; Horn *et al*., 2014). Such a possibility is supported in C_4_ clades such as the Andropogoneae grasses and the *Euphorbia* subgenus *Chamaesyce*, where a diversity of C_4_ species arose as lineages expanded into new niches following acquisition of the C_4_ pathway (Spriggs *et al*., 2014; Horn *et al*., 2014; Lundgren *et al*., 2015). Rapid species radiation following CAM evolution is documented for at least five of the *Euphorbia* CAM clades from the mid-Miocene to the Pliocene, roughly coincident with C_4_ grassland expansion across the globe and the peak of both C_4_ and CAM origins (Fig. 2). Together with the C_4_ species expansion within the subgenus *Chamaesyce*, these CAM radiations produced half of the species in the large genus *Euphorbia* (Horn *et al*., 2014). In the bromeliads, a doubling of the speciation rate was modelled to follow CAM evolution in subfamily Bromelioideae (Silvestro *et al*., 2014; but see Givnish *et al*., 2014, who did not detect this pattern across the family as a whole). Indeed, in the bromeliad lineages consisting wholly of CAM species, such as the *Dyckia* and *Hechtia* clades of xeric terrestrial succulents, and the extreme epiphytic ‘atmospheric’ forms in *Tillandsia*, it could be argued that acquisition of CAM was a prerequisite for colonization of these respective habitats (Crayn *et al*., 2004, 2015). An increased diversification rate following CAM acquisition is also documented in numerous but not all CAM orchid clades (Givnish *et al*., 2015; Bone *et al*., 2015; Li *et al*., 2019; Gamisch *et al*., 2021). In the Eulophiinae orchids, a trend towards increased diversification rate is inferred in the late Miocene following CAM acquisition (Bone *et al*., 2015). Similarly, a late Miocene to Pliocene diversification is reported for *Crassula* clades B and C, which contain known strong CAM species and well-developed succulent perennials (Bruyns *et al*., 2019; Lu *et al*., 2022). Rapid diversification of the most speciose clades of the Cactaceae and Agavoideae is also predicted to have occurred in the late Miocene to Pleistocene (Good-Avila *et al*., 2006; Arakaki *et al*., 2011; Hernández-Hernández *et al*., 2014; Jiménez-Barron *et al*., 2020). One of the most spectacular species radiations occurred in southern Africa in the core Ruschioideae of family Aizoaceae, in which lineage diversification rates 3–10 times greater than typical angiosperm rates are estimated for the late Miocene to mid-Pleistocene interval (about 8–1 Ma), when aridification was intensifying in the region (Klak *et al*., 2004, 2017).

Numerous hypotheses are proposed for the increased diversification of CAM lineages, with climate deterioration and regional drying being the common mechanism. In southern Africa, a seasonal shift to cool, somewhat moist winters is suggested to be a particularly strong cause of CAM species diversification because the physiology of CAM functions well where nights are relatively cool and humid (Lüttge, 2004; Holtum *et al*., 2016). We argue here that low CO_2_ should also be considered as a driver for diversification of CAM species, probably in concert with these other environmental changes. One interesting observation raised by Horn *et al.* (2014) is that the increased diversification rate in the CCM-clades of *Euphorbia* in the later Miocene could be due to increased establishment enabled by CAM or C_4_ photosynthesis (although CAM activity may take several months to be manifested in newly germinated seedlings of terrestrial succulents). Greater degrees of seedling establishment and plant survival could have reduced extinction rates and, by doing so, increased diversification rates. Low CO_2_ has recently been linked to reduced establishment of C_3_ seedlings, particularly in warm, dry environments (McCann and Sage, 2022). By enhancing early carbon gain and water savings, CAM could offset the deleterious effects of low CO_2_ on mortality of establishing plants and thus enhance species fitness and diversification.

## PHYSIOLOGICAL EFFECTS OF CO_2_ VARIATION ON C_3_ AND CAM PHOTOSYNTHESIS

Low atmospheric CO_2_ challenges C_3_ plants in three fundamental ways (Sage, 2013). First, below 1000 ppm, CO_2_ becomes limiting for the Rubisco carboxylation reaction of photosynthesis in its role as a substrate, with recent atmospheric values of CO_2_ falling below the *K*_M_ of Rubisco for CO_2_. At 25 °C, Rubisco *K*_M_ for CO_2_ is 8–21 µm in C_3_ plants, whilst CO_2_ concentration in the chloroplast solution is about 8 µm when atmospheric CO_2_ is 400 ppm, assuming the equilibrium gas phase ratio between chloroplast stromal CO_2_ and atmospheric CO_2_ is 0.6 (von Caemmerer and Quick, 2000). Second, Rubisco also oxygenates its substrate RuBP in the first step of photorespiration, with CO_2_ acting as a competitive inhibitor of the oxygenase reaction. With less CO_2_, particularly below 500 ppm, the suppression of Rubisco oxygenation is reduced and the O_2_ inhibition of Rubisco carboxylation increases (Ehleringer *et al*., 1991). Together, reduced CO_2_ supply as a substrate and greater photorespiration markedly reduce photosynthetic capacity of C_3_ plants during the post-Oligocene period of depleted atmospheric CO_2_ (Sage and Stata, 2015). Elevated temperature directly aggravates CO_2_ limitations by reducing the solubility of CO_2_ relative to O_2_, increasing the *K*_M_ of Rubisco for CO_2_, and enhancing oxygenase activity and photorespiratory inhibition (Sharkey, 1988). Thus, low CO_2_ effects on Rubisco activity are greatest in warm conditions where C_4_ and CAM species richness can be high (Kluge and Ting, 1978; Sage *et al*., 2018).

**Fig. 3.**
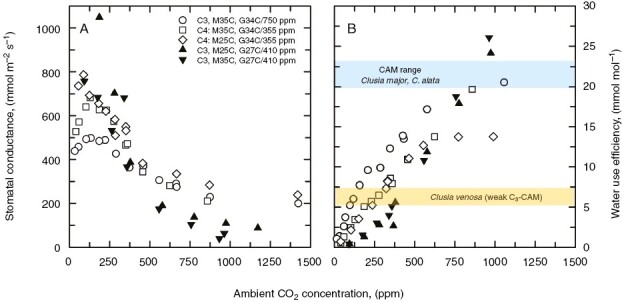
(A) The response of stomatal conductance to ambient measurement CO_2_ for a C_3_ plant (*Chenopodium album*) and a C_4_ plant (*Amaranthus retroflexus*) grown (G) at either 27 or 34 °C, and 355, 410 or 750 ppm CO_2_ as indicated, and measured (M) at 25 or 35 °C. (B) The photosynthetic water use efficiency for the responses shown in A, where water use efficiency is determined as the ratio of photosynthesis to transpiration determined using leaves in a gas exchange chamber. Reprinted from Sage and Stata (2021), with results from Sage *et al.* (1995), where growth conditions and gas exchange measurement procedures are described. *Clusia* values are from Lüttge (2007*b*).

The third fundamental limitation imposed by reduced CO_2_, and the one perhaps most relevant to CAM evolution, is the aggravation of water deficiency. C_3_ plants respond to low atmospheric CO_2_ by opening stomata, thus increasing stomatal conductance (*g*_s_) to water-vapour diffusion (Fig. 3A; Sage and Coleman, 2001). As *g*_s_ increases with declining CO_2_, transpiration rates rise and water use efficiency (WUE) of photosynthesis declines (Fig. 3B). This creates two potential threats to plants, which are most acute in hot, dry environments. In the short term, rapid rates of transpiration can reduce leaf water status (declining water potential and cell turgor), potentially to the point of tissue injury unless water supply is sufficient and the hydraulic transport capacity of the plant can deliver water to the leaves as fast as it evaporates (Schulze and Hall, 1982; Kocacinar and Sage, 2004; Osborne and Sack, 2012). High vapour pressure difference (VPD) between leaf and air directly enhance transpiration, which may bring the benefit of transpirational cooling if water supply is adequate, but which increases the probability of leaf wilting and injury when water availability is restricted. High VPD occurs in warmer, drier climates of low humidity, particularly in hot midday conditions (Schulze and Hall, 1982). Over the long term, enhanced *g*_s_ at reduced atmospheric CO_2_, particularly at high VPD, will promote elevated transpiration rates that more rapidly consume soil water and raise the risk of crippling water deficit. Consistent with this reasoning, soil water deficits are widely documented to be more common in low relative to high atmospheric CO_2_ treatments (Polley *et al*., 1997; Morgan *et al*., 2004, 2011). As drought intensifies, plants close stomata to conserve water, but this aggravates CO_2_ deficiency in low atmospheric CO_2_ (Sage and Coleman, 2001; Gerhart and Ward, 2010; Franks *et al*., 2013). For this reason, selection for CCMs is considered to have been particularly strong in warm, dry climates of reduced atmospheric CO_2_ (Sage and Stata, 2015).

Plants have a number of mechanisms to compensate for the combination of low CO_2_, drought and heat (which elevates VPD). To sustain high transpiration rates without turgor loss and potential leaf injury, plants can invest in proportionally greater fractions of root mass and hydraulic conductivity in stems and leaves than they might when water is abundant (Sage, 2001; Osborne and Sack, 2012; Griffiths *et al*., 2013). At the leaf level, this could mean a denser network of veins and larger bundle sheath cells, facilitating the rise of photorespiratory glycine shuttling in hot environments (Sage, 2001; Osborne and Sack, 2012; Christin *et al*., 2013; Griffiths *et al*., 2013). An alternative compensation mechanism is to form water storage tissue, increasing tissue succulence and resilience to episodic water deficiency (Gessner, 1956; Ogburn and Edwards, 2010; Griffiths and Males, 2017). In many species, succulence occurs in heterotrophic tissues of roots and stems, or non-photosynthetic hydrenchyma of leaves, leaving the photosynthetic tissue little changed and C_3_ in structure and function (Barrera Zambrano *et al*., 2014; Borland *et al*., 2018). However, if the photosynthetic chlorenchyma becomes succulent, the possibilities for altered photosynthetic function may arise because a larger cell and vacuolar volume allows greater metabolite storage per unit surface area of leaf tissue (Smith *et al*., 1996). In this manner, succulence of photosynthetic cells for drought resistance may become an exaptation for the initiation of CAM function. As high vein density may predispose C_4_ evolution in response to the combination of drought and low CO_2_, succulence of photosynthetic tissues may predispose C_3_ plants to evolve CAM (Sage, 2002; Edwards and Ogburn, 2012; Edwards, 2019; Heyduk *et al*., 2016; Luján *et al*., 2022). Moreover, as succulence increases, the large photosynthetic cells become more densely packed, which could impede diffusion of CO_2_ into the tissue (Maxwell et al., 1997; Nelson *et al*; 2005). In this manner, succulence itself could aggravate CO_2_ deficiencies and become a contributing factor for CAM evolution.

**Fig. 4.**
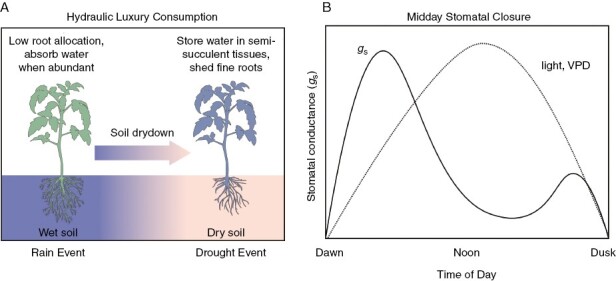
A schematic outlining two key drought strategies in C_3_ plants that may facilitate the initiation of CAM evolution. (A) Outline of the function of hydraulic luxury consumption, where water is absorbed by a plant when abundant and maintained in storage tissues when the soil dried, thereby extending the growing season and/or enhancing survival during severe drought. (B) Strong midday stomatal closure is shown (solid line), where stomatal conductance (*g*_s_) serves as an index of stomatal aperture. Typical patterns of light intensity and vapour pressure difference (VPD) between leaf and air are shown over the course of the day. Midday stomatal closure profiles are idealized patterns based on responses in Schulze and Hall (1982).

One of the important findings from research on the CO_2_-response of C_3_ plants is a marked reduction in root growth in low CO_2_ treatments (<400 ppm), due to both slower growth and reduced allocation to root relative to shoot biomass (Sage, 1995; Campbell and Sage, 2002; Gerhart and Ward, 2010; Bond and Midgley, 2012). Reduced root allocation aggravates drought limitations by reducing a plant’s ability to acquire water, which in turn restricts *g*_s_. Because roots are expensive to make and maintain, the relative cost of water acquisition should rise in low-CO_2_ atmospheres, potentially leading to stronger selection for other mechanisms that reduce water costs. C_3_ plants can reduce relative water costs through two important strategies – reducing the cost of water acquisition and reducing the cost of water consumption (Fig. 4). Reducing water acquisition costs can be achieved through luxury consumption of water, where water is absorbed when it is abundant and cheap, and stored for later use when external supplies are scarce (Fig. 4A; Chapin *et al*., 1990). The concept of luxury consumption generally refers to nutrient acquisition and storage (Lambers *et al*., 2008), but can equally well describe acquisition and storage of water when it is available at low cost. With luxury consumption comes a need for water storage tissue, which can be achieved in a number of ways involving roots, woody tissue or the mesophyll cells of leaves. When water storage arises in non-photosynthetic tissues, it is unlikely to enhance CAM possibilities and may even act as an impediment. When it occurs in photosynthetic tissues, then it can readily become an exaptation for CAM.

**Fig. 5.**
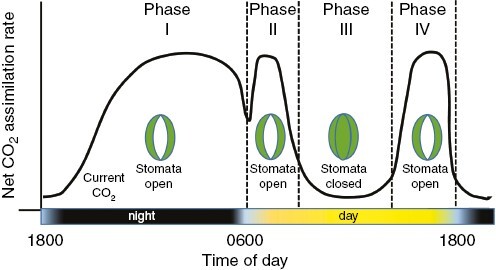
Idealized phases of CO_2_ assimilation exhibited by a strong CAM plant during a 24-h diurnal cycle, as described by Osmond (1978). Note that obligate CAM plants often exhibit an essentially biphasic cycle, with only phases I and III being apparent. The diagram closely follows Fig. 10.2 in Borland *et al.* (2018).

A second mechanism to reduce water costs is to minimize water use when the cost of transpiration is high. This may mean specialization for growth in the cooler, wetter seasons of the year, as seen in desert ephemerals and drought-deciduous trees and shrubs (Smith *et al*., 1997). For plants active in hot, dry locations, or in warm climates with limited soil, water costs can be reduced by greater degrees of midday stomata closure (Schulze and Hall, 1982; Lüttge *et al*., 1986). As outlined in Fig. 4B, midday stomata closure is the phenomenon where *g*_s_ is greater early and late in the day when VPD is low and transpiration potential is reduced, and then reduced at midday when VPD is high. In plants with strong midday stomatal closure, the stomatal rhythm approximates stomatal rhythms in CAM during daytime phases (compare Figs 4B and 5). We hypothesize that if such a pattern were to become constitutive in a C_3_ plant, perhaps by linkage to a circadian regulator or via increased sensitivity to abscisic acid (ABA), calcium signals and VPD, then midday stomatal closure could also become a precursor for CAM by facilitating eventual establishment of the CAM stomatal rhythm (Hotta *et al*., 2007; Males and Griffiths, 2017*a*, *b*; Kamrani *et al*., 2022). Control of circadian oscillators over stomatal rhythms and WUE has been established in *Arabidopsis* (Simon *et al.*, 2020; Kamrani *et al.*, 2022), suggesting alteration of midday stomatal depression by circadian control is a possibility worth examination.

### Experimental responses of CAM photosynthesis to CO_2_ variation

**Fig. 6.**
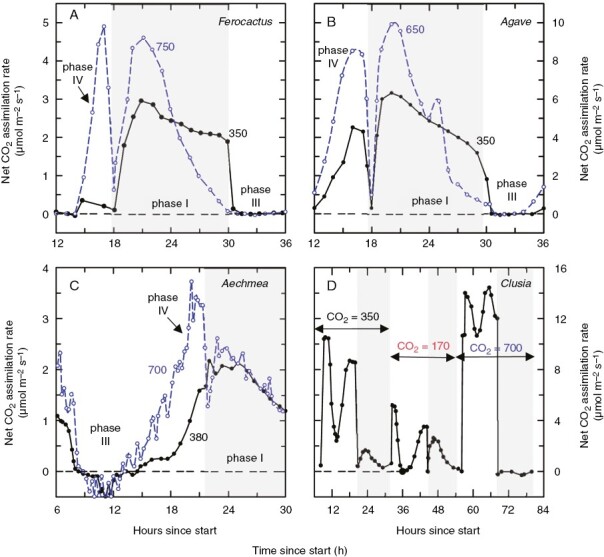
The diurnal cycle of net CO_2_ assimilation rate in select CAM plants measured at recent and elevated levels of atmospheric CO_2_. In A–C, plants were grown and measured at either recent (black lines and filled symbols) or elevated CO_2_ (blue lines and open symbols), with measurement CO_2_ in ppm given above each curve. Grey shading indicates nocturnal periods. In D, plants were grown at 350 ppm CO_2_ and measured at the CO_2_ values indicated in the panel above the double-headed arrows. (A) *Ferocactus viridescens* (originally published under the synonym *Ferocactus acanthodes*) grown and measured at 350 or 650 ppm (Nobel and Hartsock, 1986); (B) *Agave deserti* grown at 350 or 650 ppm CO_2_ (Nobel and Hartsock, 1986); (C) the CAM bromeliad *Aechmea* ‘Maya’ grown and measured at 380 or 700 ppm (Ceusters *et al*., 2008); (D) the flexible CAM hemiepiphyte *Clusia uvitana* grown at 350 ppm CO_2_ and measured at either 170, 350 or 700 ppm as indicated in the panel (Winter *et al*., 1992). Note in A–C that phase IV is enhanced by high CO_2_ relative to the lower CO_2_ response; while in A–C, phase I is enhanced early in the dark period but not later. In D, note how elevated CO_2_ eliminates dark CO_2_ fixation, after enhancing daytime CO_2_ fixation, while at 170 ppm CO_2_ the daytime fixation rate is much less than at higher levels of CO_2_, whereas the nighttime fixation rate is higher.

While numerous studies have investigated CAM plant responses to elevated atmospheric CO_2_ concentrations predicted for the next century (Winter *et al*., 1997; Drennan and Nobel, 2000; Ceusters and Borland, 2010; Pereira *et al*., 2021; Sage and Stata, 2021; Hultine *et al*., 2023), only a few have examined responses of CAM plants to CO_2_ atmospheres below recent historical concentrations (Winter *et al*., 1992, 2014). This does not appear to be a major impediment to evaluation of interactions between declining CO_2_ and CAM evolution, because most CAM origins appear to be Miocene to Pliocene in age, when CO_2_ was similar to current atmospheric levels near 400 ppm. The research with CAM plants in very low CO_2_ (170–280 ppm) is notable in that it shows the relative contribution of nocturnal CO_2_ uptake by CAM to 24-h CO_2_ fixation is markedly enhanced relative to what is observed at higher CO_2_ levels (Fig. 6D; Winter *et al*., 1992, 2014).

CAM plants are widely noted to perform better at elevated CO_2_, with improvements in carbon gain and growth that often exceed that of C_3_ species (Cui *et al*., 1993; Cui and Nobel, 1994; Graham and Nobel, 1996; Winter *et al*., 1997; Zhu *et al*., 1999; Drennan and Nobel, 2000; Zotz *et al*., 2010, 2023; Wagner and Zotz, 2018). This observation could lead to a conclusion that high CO_2_ enhances CAM photosynthesis such that low CO_2_ would not be necessary for CAM evolution. However, the improvement of CAM plant performance in elevated CO_2_ is due more to increased C assimilation during the C_3_ phases of the diurnal CAM cycle and, for facultative CAM plants, increased C gain when the plants are operating in the C_3_ mode (Fig. 6; Winter *et al*., 1997, 2014; Zhu *et al*., 1999; Ceusters and Borland, 2010; Zotz *et al*., 2023). Responses of four representative CAM plants are shown in Fig. 6. In each, there is a pronounced stimulation of daytime CO_2_ assimilation, mostly in phase IV. Notably, phase IV can appear in obligate CAM plants that do not normally exhibit it when they are exposed to reduced CO_2_ (Fig. 6A; Graham and Nobel, 1996). Phase I usually, but not always, exhibits enhanced C gain in elevated CO_2_ (Drennan and Nobel, 2000). Often, phase I CO_2_ uptake in elevated CO_2_ is enhanced early in the dark period relative to rates observed in plants at lower CO_2_ concentration, but then drops below CO_2_ assimilation rates of plants in low CO_2_ later at night (Fig. 6A, B). This response is interpreted to result from earlier depletion of carbohydrate reserves needed for glycolytic production of PEP, and/or the vacuole being filled to capacity with malic acid earlier in the dark period, such that CO_2_ assimilation becomes restricted earlier in the dark period in plants at elevated CO_2_ (Drennan and Nobel, 2000). In bromeliads with smaller cell vacuoles, stimulation of phase I CO_2_ uptake by elevated CO_2_ is negligible (Drennan and Nobel, 2000; Ceusters *et al*., 2008). Based on these observations, we hypothesize that mildly succulent C_3_ and incipient C_3_ + CAM species would be limited in their ability to accumulate malic acid at night, such that any CAM contribution at high CO_2_ would be a small fraction of the daily C gain. By contrast, in low CO_2_, the relative CAM contribution could be much higher, allowing for a stronger signal that selection could act upon, and thus favour the strengthening of CAM activity at night.

On the water side, a major fitness attribute that CAM confers is very high WUE, typically 3–10 times that of C_3_ photosynthesis in recent atmospheres (Kluge and Ting, 1978; Osmond 1978; Nobel, 1988; Lüttge, 2004, 2007*a*, *b*; Borland *et al*., 2009). Elevated CO_2_ enhances WUE in C_3_ plants through the combination of enhanced C assimilation and reduction in stomatal conductance (Sage, 1994; Ainsworth and Long, 2005; Venter *et al*., 2022). Figure 3B shows the effect of rising CO_2_ on WUE for a widely distributed C_3_ weed, *Chenopodium album*, grown at 380 ppm and elevated CO_2_ levels of 750 ppm. As the measurement CO_2_ in the leaf cuvette increases to double historical values, the WUE of *C. album* grown in either current CO_2_ or enhanced CO_2_ rises 3- to 4-fold, to approach values exhibited by CAM *Clusia* species (Fig. 3B). C_3_*Clusia* species exhibit similar WUE values to *C. album* grown and measured at recent historical values of atmospheric CO_2_ (Lüttge, 2007*b*). This evidence shows that in elevated CO_2_, CAM-like WUE values are possible in the C_3_ flora such that there may be a reduced need for CAM to improve the water economy. Hence, the selection pressure for CAM based on water conservation alone should have been much less in a high CO_2_ atmosphere.

## C_3_ + CAM VERSUS STRONG CAM EVOLUTION IN HIGH CO_2_

If elevated CO_2_ reduces the need for strong CAM to enhance plant fitness, then why, and how, might weak C_3_ + CAM physiologies evolve in a high CO_2_ world as indicated by the pre-Miocene appearance of C_3_ + CAM in a number of lineages such as the Crassulaceae and Portulacineae? While the possibilities require focused research using sister C_3_ and C_3_ + CAM species, for now we provide a hypothesis. In elevated atmospheric CO_2_, drought and other relevant stresses would still have occurred although with less frequency and intensity than at reduced CO_2_, assuming identical moisture regimes. As defined by Winter (2019), weak CAM cycling contributes little to plant C balance and thus is not an important component of growth, reproduction and competitive ability. Instead, by recycling respired carbon, CAM cycling serves as a maintenance mechanism during extremes of drought or salinity, which occurs in dry climates and dry microsites such as rock outcrops and tree branches, even at high CO_2_ (Griffiths, 1989; Martin, 1996; Pilon-Smits *et al*., 1996; Holtum *et al*., 2016). The ability to use CAM to recycle respiratory CO_2_ at night can substantially delay C depletion and allow a plant to survive dry episodes with completely closed stomata. In this regard, a weak CAM cycle and any associated succulence could serve as a survival mechanism, conferring fitness regardless of the prevailing CO_2_. Moreover, the costs of weak C_3_ + CAM physiology and structure are probably low compared to strong CAM, both in an absolute sense and in terms of how they might interfere with C_3_ photosynthesis (Edwards, 2019). Compared to strong CAM plants, succulence of weak CAM plants tends to be low, mesophyll cells are not tightly packed, investment into the C_4_ enzymes is small and the energy costs of CAM function are minimal (Edwards, 2019; Luján *et al*., 2022; Leverett *et al*., 2023). As these costs rise with the evolution of stronger CAM phenotypes, the benefits should also increase, but in high CO_2_, C_3_ plants already experience the benefits of high WUE and carbon gain without the cost.

**Fig. 7.**
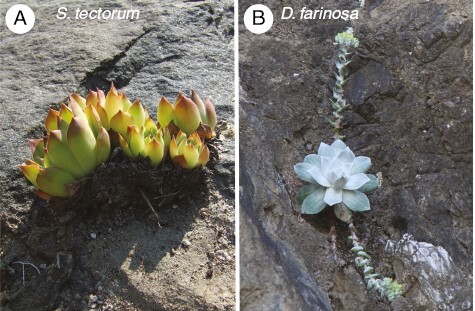
Two Crassulaceae lithophytes growing on rock faces. (A) *Sempervivum tectorum* growing on rock outcrops near Gravenhurst, Ontario, Canada; and (B) *Dudleya farinosa* growing on a cliff face along the Mendocino coast of California, USA (lat. 40.28, long. −124.36). The photos demonstrate examples of where strong CAM may have arisen in high-CO_2_ environments of the pre-Miocene. We hypothesize strong CAM evolution may have occurred in elevated CO_2_ on rocky substrates such as these shown, where water deficiency could have been so frequent and intense as to select for persistent stomatal conservatism, low internal CO_2_ and, possibly, CAM intensification.

Given these considerations, how might strong CAM evolve in elevated CO_2_ atmospheres as suggested by certain Crassulaceae clades? For strong CAM to evolve in elevated CO_2_, we hypothesize that it would occur when plants experience chronically low internal CO_2_ concentrations and C deficiency brought about by persistently low conductance to CO_2_ diffusion. Persistently low conductance would be most evident in warm environments with high VPD and restricted water supply (Cernusak *et al*., 2013; Mok *et al*., 2023), such as the soil-free lithophyte habit on rock outcrops and cliff-faces. Consistent with this hypothesis, many modern Crassulaceae species are lithophytes specializing in survival on rock surfaces with minimal soil (Fig. 7), leading us to hypothesize that the lithophyte habit in Crassulaceae is also ancient, pre-dating low atmospheric CO_2_. If so, they may have relied on extreme water conservation measures that facilitated the transition from weak to strong CAM in elevated atmospheric CO_2_ (see Males and Griffiths, 2017*b*, for a discussion of stomatal control in lithophytic bromeliads). These considerations highlight the probability that low CO_2_ is not an absolute requirement for strong CAM evolution, but rather, a condition that increases probabilities for CAM origin, particularly in combination with other stresses such as heat and drought.

## SYNTHESIS – DID LOW CO_2_ PROMOTE CAM ORIGINS?

The evidence presented here supports a role for low CO_2_ in facilitating the evolution of strong CAM photosynthesis and, for many lineages, C_3_ + CAM physiologies as well. Weak CAM appears to have arisen in some lineages during elevated CO_2_ episodes prior to the Oligocene, which could be consistent with its role in enabling plant survival during episodic drought extremes. Such use of weak CAM is widely considered to facilitate evolution of stronger CAM modes, by establishing the biochemical and regulatory capacity for limited CAM activity, which could then be upregulated (Pilon-Smits *et al*., 1996; Sage, 2002; Bräutigam *et al*., 2017; Winter and Smith, 2022). However, this presents a conundrum. If it were a simple matter of upregulation of weak CAM to produce strong CAM, then why is there not widespread evidence for strong CAM physiology dating well into the Oligocene or Eocene when CO_2_ was elevated? One possibility is that weak CAM was uncommon at elevated CO_2_, and hence there was little opportunity for strong CAM to evolve. The relatively few C_3_ + CAM clades postulated for the Eocene support this case. Another possibility is there were significant constraints to evolving strong CAM, such that strong and consistent selection pressure was needed to surmount these constraints. Edwards (2019) argues that acquisition of pronounced succulence may have been the evolutionary barrier to strong CAM evolution. If so, then surmounting this barrier may have required the strong selection pressure created by low atmospheric CO_2_, or a particularly harsh set of circumstances as may have occurred on dry rock outcrops in a warm, high-CO_2_ world.

A second consideration is that CAM is widely regarded as a drought-tolerance mechanism, and in many regions where CAM species arose and diversified, intensification of aridity in the past 35 million years corresponds with the appearance of the CAM clades (Keeley and Rundel, 2003; Bone *et al*., 2015; Li *et al*., 2019; Heyduk, 2022). Aridification is generally considered a primary driver for CAM evolution, with low CO_2_ generally being ignored or mentioned in passing (but see Arakaki *et al*., 2011; Heyduk, 2022). If drought were the predominant driver, then why did strong CAM not commonly arise in older arid habitats that pre-date the Oligocene? The most common dry environment of the pre-Miocene world would have been the epiphytic habits of the widespread tropical and subtropical forests of Eocene and Oligocene landscapes; yet, as indicated in Fig. 1, there is little evidence for ancient origins of strong CAM epiphytes prior to 30 Ma. [Tropical forests have existed since pre-Cretaceous time, although dominated by gymnosperms until the mid- to late Cretaceous when angiosperms asserted their dominance (Jaramillo, 2023). The epiphytic niche probably pre-dated the angiosperms, with ferns occupying this habitat as they do now in temperate rainforests. The modern type of angiosperm-dominated rainforest became widespread by the Eocene (Jaramillo, 2023), and hence the epiphytic niche in its modern form should have been present before epiphytic CAM lineages appeared. A critical event in development of the rainforest in its modern form was the establishment of habitat where many lineages that later evolved CAM would arise, thus meeting a precondition (compatable taxa) for later CAM evolution.]

Strong CAM origins in the tropical epiphytes such as the bromeliads, orchids and *Hoya*–*Dischidia* clades are clearly Miocene or later, when reduced CO_2_ is evident. Extensive radiation of terrestrial CAM plants in southern Africa is also evident in the later Miocene and Pliocene, despite semi-arid to arid landscapes being present since the Cretaceous in Namibia (Ward *et al*., 1983). Aridity in the Atacama region of South America is also ancient, extending back 150 Ma (Hartley *et al*., 2005). We argue that low CO_2_ may have made the difference in the evolutionary emergence of CAM, and should therefore be considered a primary environmental driver, along with drought.

As we move forward, rising CO_2_ should remove an important facilitating agent for CAM success, and it is possible that CAM species will become restricted and outcompeted by C_3_ plants. In epiphytic floras, for example, more aggressive growth of C_3_ species in elevated CO_2_ might be expected to crowd out CAM species, particularly since many of the CAM plants use obligate CAM with weak C_3_ phases; however, the evidence for this possibility is not strong (Zotz *et al*., 2023). Elevated CO_2_ is unlikely to harm CAM species directly, given their flexibility and propensity to rely increasingly on C_3_ metabolism in higher CO_2_, and one might hypothesize CAM being able to move to more extreme epiphytic microsites as their WUE improves. Before any of this happens, however, there are far more serious threats to the CAM flora that must be addressed, namely from habitat destruction, run-away fire cycles, over-harvesting of CAM plants for horticultural uses and invasive species outbreaks (Grace, 2019; Sage and Stata, 2021; Zotz *et al*., 2023; Hultine *et al*., 2023). CAM photosynthesis is not unique in feeling the heat of anthropogenic global change, as the C_4_ flora and much of the C_3_ flora are under similar threats (Sage, 2020). Because so much of the world’s biota is currently threatened, there may be too many critical needs to prioritize conservation of CAM plants; however, the CAM flora has many human friends in the numerous orchid, bromeliad and succulent societies around the world. Conservation biologists should collaborate with these enthusiasts to ensure CAM diversity is protected as global change intensifies.

To close, we are encouraged by the extensive phylogenetic progress of the past 10–20 years which made the age estimates presented here possible. These estimates will undoubtedly be refined as comprehensive studies add new CAM taxa and character states to increasingly detailed phylogenies. While age estimates of CAM will improve, an important additional benefit will be a greater understanding of how CAM was assembled from C_3_ ancestors, allowing CAM to become one of the powerful models for understanding complex trait evolution in general. One of the key challenges to developing a more comprehensive understanding of CAM evolution will be the phenotyping of CAM character states. As a first pass, we recommend continued work to survey δ^13^C values of bulk leaf tissue to distinguish strong CAM phenotypes from C_3_ and C_3_ + CAM phenotypes. Surveys of δ^13^C can exploit large collections of plants in the world’s herbaria, enabling comprehensive representation across a phylogeny. To better resolve potential CAM species, δ^13^C values could be determined on starch and sugars extracted from herbarium specimens. Leaf starch reflects C gain over the prior day or two, so it would be less prone to dilution of the δ^13^C signal by C_3_ photosynthesis that predominated early in development or before CAM is induced in facultative CAM plants. Within the group of species with C_3_-like δ^13^C values, follow-up physiological assessments such as of diurnal acid cycling could be used to further screen for CAM, using phylogenies of C_3_ to CAM lineages to strategically target transitional species. Physiology studies will require living plants, which may turn out to be the greatest challenge, given the cost, regulatory restrictions and in some places danger of collecting species. Where the challenge is great, so can be the opportunity. An excellent way to gain access to plant species is to build relationships between diverse groups of colleagues from across the globe. As Klaus Winter has demonstrated throughout his career (Holtum, 2023), such relationships should not simply be a means to acquire plants, but also a way to build networks of expertise by forming collaborations with colleagues and their students in regions rich with CAM diversity. While CAM research immediately profits through such networks, the ultimate benefit may be the establishment of local experts who will lead efforts to preserve Earth’s CAM flora. The investment by Klaus Winter into this human side of the CAM adventure may turn out to be one of his greatest legacies.

## SUPPLEMENTARY DATA

Supplementary data are available at *Annals of Botany* online and consist of the following.

Appendix S1: Sources and assumptions for dating the CAM lineages presented in Fig. 1B and C.

mcad122_suppl_Supplementary_Appendix
