# Supplementary material for: Atmospheric CO2 decline and the timing of CAM plant evolution
Source: Ann Bot. 2023 Aug 29;132(4):753–70. doi: 10.1093/aob/mcad122 (PMC10799994; doi:10.1093/aob/mcad122)
Supplement: mcad122_suppl_Supplementary_Appendix [file mcad122_suppl_supplementary_appendix.pdf]

ONLINE SUPPLEMENTAL APPENDIX S1

Sources and Assumptions for Dating the CAM Lineages Presented in Figure 1B and 1C.

To generate Figure 1 panel 1B, we report dates from phylogenetic chronograms for stem and crown nodes of CAM-specific clades. The identification of CAM specific clades was based on densely-sampled $\delta^{13}C$ surveys, gas exchange analyses, or diurnal acid accumulation, which were then mapped onto phylogenies reported below. For lineages where the CAM status of sister clades are not known, we assumed they were C_3 if not listed as CAM by Gilman *et al*. (2023), with the notable exception of the Crassulaceae where uncertainty in CAM distribution led us to not have confidence in this assumption. As Gilman *et al*. note, follow-up surveys of sister clades should be conducted as prior work did not always report non-CAM determinations, or did not survey sister clades if they lacked strong succulence.

For Fig. 1C, we used Gilman *et al.* (2023) and Smith and Winter (1996) to identify clades containing some CAM species, and use dated phylogenies for divergence dates of these clades. Generally, CAM determinations for species within these clades are limited to a handful of species, so it is not possible to identify CAM specific clades, nor even when CAM may have arisen. Therefore, the date of the divergence given are for the clade in which the limited number of CAM species occur. This approach generally will not identify CAM age in the clade, but does set the oldest boundary for CAM. Generally, CAM will be much younger than the clade age.

What follows is a listing of clades examined and the source of the phylogenetic dates. For each clade, see the Fig. 1 legend for the clade names corresponding to each histogram as given in the sources listed below. The order of presentation matches that shown in Fig. 1B and 1C.

We note that our survey identified 73 CAM lineages, in contrast to the minimum of 66 CAM lineages noted by Gilman *et al*., (2023). This discrepancy reflects different assumptions and authorities in the two studies, and the preliminary nature of the respective efforts given the incomplete knowledge of CAM diversity and phylogenetics within many CAM families. Rather than harmonize the two estimates, we chose to stay with our estimate of 73 lineages to recognize that identification of CAM lineages is an ongoing area of research.

Figure 1B (listed bottom to top within the panel):

Dendrobium (Orchidaceae), CAM clades 1-8 in Table 2 from Li *et al.* (2019).

Eulophiinae (Orchidaceae), CAM clades 1-4 in Table 2 (Bone *et al.,* 2015).

Bulbophyllum from Gamisch *et al*. (2021).

Bromeliads from the Discussion in Crayn *et al*. (2015) in the order of the clades Hectioideae, *Dyckia*, *Puya*/Core Bromeliads, core *Tillandsia*, and *Tillandsia utriculata. Tillandsia* clade dates are estimated from Givnish *et al.* (2014).

Agave clades are *Hesperaloe*, the crown *Yucca*, and *Agave* clades as delineated by Heyduk et al. and dated by McCain et al. (McKain *et al*., 2016; Heyduk *et al*., 2022).

Euphorbia clades follow Table 2 in Horn et al. (2014), where the histograms of Fig. 1B represent 15 of their 17 postulated strong CAM clades, as listed in the Fig. 1 legend. Anthacanthae + *Balsamis* was assumed here to represent one clade, Anthacanthacae clade 8.

Aizoaceae: The three CAM clades from bottom to top in Fig. 1B are *Tetragonia*, Mesembyranthoideae, and core Rushoideae. Divergence dates for these clades follow Klak *et al.* for the *Tetragonia* clade, and Liede-Schumann et al. for the other two clades (Klak *et al.,* 2017; Liede-Schumann *et al.* 2020). To estimate the divergence of these strong CAM clades we assumed the sister clades were either C_3+CAM or C_3, following their absence from the CAM genera listed by Gilman *et al.* (2023).

Portulacineae: The crown node of the Portulacineae is thought to be C_3+CAM, given the distribution of C_3+CAM character states in the clades that branch at distal nodes in the phylogeny. Strong CAM evolved multiple times in the individual clades of Portulacinae shown in Fig. 1B. Stem and crown node dates follow Wang et al. and Arakaki et al. for the Portulacineae and its CAM-specific clades *Alluadia* + *Allauadiopsis*, core Cactoideae, *Grusonia*, and a clade of *Tephrocactus* and *Opuntia* (Arakaki *et al.* 2011; Wang *et al.,* 2019). Stem and crown node dates for strong CAM in the *Anacampseros* are from Ocampo and Columbus (2010) following CAM determinations by Guralnik *et al*. (2008).

Rubiaceae: Two lineages of epiphytic ant-plants in the tribe Psychotrieae of Rubiaceae are dated from Fig. 1 in Chomicki and Renner (2016). *Myrmecodia beccarii*, which occurs in northern Queensland, Australia, is maximum 3.6 Ma old. Three CAM species in *Squamellaria* endemic to Fiji are estimated at 1.6 Ma.

Zygophyllaceae: *Bulnesia retama* in the subgenus Larreiodeae is recently shown to be the only C_3+ CAM species in the family (Mok *et al.,* 2023). It splits from its sister species at 1 (crown) to 4 (stem) Ma (Böhnert *et al*., 2020).

Figure 1C: Clades containing CAM species, but CAM position uncertain.

Pyrrosia ferns follow Wei *et al.* (2017) who conclude CAM likely evolved in a distinct Austral-Asian clade with deeply sunken stomata at 17.98 Ma.

Peperomia dates are from the average of crown dates in Nauman *et al.* (2013) and Massoni et al. (2015). The ancestral condition of *Peperomia* appears to be C_3, with CAM arising multiple yet uncertain times at more distal nodes in the *Peperomia* tree.

Asphodelaceae (*Aloid* clades): The origin of the strongly succulent and largely CAM clade *Aloe* and relatives (=genera in the Aloiod clade which includes *Aloidendron* and *Haworthia*) is based on Additional Files 2 and 3 in Grace *et al.* (2015). We presumed the crown of *Bulbine* + Asphodeloideae indicates when CAM evolved in this clade.

Orchidaceae clades are estimated by mapping isotope data and CAM clades from the undated phylogeny of Silvera *et al*. (2009, 2010; see also Silvera *et al*., 2005) onto the dated supermatrix

tree with all 301 taxa in the supplement of Givinish *et al.* and using the latter's stem dates to estimate clade age (Silvera *et al.* 2009, 2010; Givnish *et al.,* 2015). Clades are 1, Maxillariinae; 2, Stanhopeinae; 3, Oncidiinae; 4, Laeliinae; 5, Vandeae; 6, Pleurothallidae. See Fig. 1B for CAM origins in *Dendrobium*.

Ottelia in the Hydrocharitaceae follows divergence dates presented in Li et al. (2019).

Pelargonium divergence is estimated by van der Kerke *et al.* (2019). Because the CAM clade is subtended within the genus, we used their crown node date.

Coleus dates are for the succulent, CAM containing clade B (Paton *et al.,* 2018).

Apocynaceae: Four independent CAM clades are apparent in the Apocynaceae (*Ceropegia, Cynachrum, Dischidia-Hoya,* and *Pachypodium*; Gilman *et al.,* 2023). Dates for divergence of the *Ceropegia* clade follows Bruyns et al. and Liede-Schumann *et al.* for the *Dischidia-Hoya* clade (Bruyns *et al.,* 2015; Liede-Schumann *et al.,* 2022). Date estimates for the *Cynachrum* divergence, and the *Pachypodium* divergence were estimated by taking their respective crown node length presented in Wang *et al*., dividing by the stem node length of the *Dischidia-Hoya* clade, and then multiplying these relative lengths times the stem node ages of the *Dischidia-Hoya* clade. This method was able to reproduce the stem node age of the *Ceropegia* clade in Bruyns *et al.* (Bruyns *et al.,* 2015; Wang *et al.,* 2023).

Asteraceae tribe Senescioneae: Three succulent clades with confirmed CAM are present in the Asteraceae – the Gynuroid clade with multiple CAM genera (for example, *Kleinia, Curio* and *Senecio meuselii*), the *Caputia* clade, and the *Othonna/Crossothonna* clade. The divergence dates of these clades are based on the ITS/ETS chronogram presented in Fig. 5 of Pelser *et al.* (2010). Other succulent Senecioneae not shown (e.g *Pictocaulon*; Cicuzza *et al*., 2017) branch at nodes that are less than 5 Ma but the presence of CAM requires confirmation.

Clusia: Late-Miocene *Clusia* divergence dates are based on Ruhfel *et al.* (2016). Lujan *et al.* (2022) show strengthening of CAM across the *Clusia* phylogeny following its divergence.

Curcurbitaceae: CAM divergence of two separate CAM-containing clades, *Seyrigia* and *Xerosicyos,* are dated in the Fig. 2 and Fig. A1 chronograms of Guo *et al.* (2020).

Gesneriaceae: Two CAM lineages are identified in the African violet family, one in the Ramondinae (*Haberlea/Ramonda*) clade, and a second in the *Cadonanthe* clade. Petrova et al. date the Ramondinae spilt from a non-CAM sister clade at a crown age of 24.5 Ma, and a stem of 30.5 Ma while Roalson and Roberts date the CAM-inclusive *Codonanthe-Nematanthus* clade to diverge at 14 to 15 Ma (Petrova *et al.,* 2015; Roalson and Roberts, 2016). However, the known CAM clade of *Codonanthe* appears younger, diverging 3 to 6 Ma. This clade is shown to have weak CAM (Guralnick *et al.,* 1986).

Crassulaceae: Dates are based on Messerschmid et al. (2020) for Crassulaceae and *Crassula* divergence, as discussed in the text. Uncertainty in phylogenetic dating and CAM occurrence lead us to show the bar for Crassulaceae fading in color towards the left of the plot.

Literature Cited

Arakaki M, Christin P-A, Nyffeler R, *et al.* 2011. Contemporaneous and recent radiations of the world's major succulent plant lineages. *Proceedings of the National Academy of Sciences of the United States of America* **108**: 8379–8384.

Böhnert T, Weigend M, Merklinger FF, Quandt D, Luebert F. 2020. Historical assembly of Zygophyllaceae in the Atacama Desert. *Frontiers of Biogeography* **12.3** e45197.

Bone RE, Smith JAC, Arrigo N, Buerki S. **2015**. A macro-ecological perspective on crassulacean acid metabolism (CAM) photosynthesis evolution in Afro-Madagascan drylands: Eulophiinae orchids as a case study. *New Phytologist* **208**: 469–481.

Bruyns PV, Klak C, Hanáček P. **2015**. Recent radiation of *Brachystelma* and *Ceropegia* (Apocynaceae) across the Old World against a background of climatic change. *Molecular Phylogenetics and Evolution* **90**: 49–66.

Chomicki G, Renner SS. **2016**. Evolutionary relationships and biogeography of the ant-epiphytic genus *Squamellaria* (Rubiaceae: Psychotrieae) and their taxonomic implications. *PLoS ONE* **11**: e0151317.

Cicuzza D, Stäheli DS, Nyffeler R, Eggli U. **2017**. Morphology and anatomy support a reclassification of the African succulent taxa of *Senecio* s.l. (Asteraceae: Senecioneae). *Haseltonia* **23**: 11–26.

Crayn DM, Winter K, Schulte K, Smith JAC. **2015**. Photosynthetic pathways in Bromeliaceae: phylogenetic and ecological significance of CAM and C_3 based on carbon isotope ratios for 1893 species. *Botanical Journal of the Linnean Society* **178**: 169–221.

Gamisch A, Winter K, Fischer GA, Comes HP. **2021**. Evolution of crassulacean acid metabolism (CAM) as an escape from ecological niche conservatism in Malagasy *Bulbophyllum* (Orchidaceae). *New Phytologist* **231**: 1236–1248.

Gilman IS, Smith JAC, Holtum JAM, Sage RF, Silvera K, Winter K, Edwards EJ. 2023. The CAM lineages of planet Earth. *Annals of Botany* (submitted, this volume).

Givnish TJ, Barfuss MHJ, Ee BV, *et al.* 2014. Adaptive radiation, correlated and contingent evolution, and net species diversification in Bromeliaceae. *Molecular Phylogenetics and Evolution* **71**: 55–78.

Givnish TJ, Spalink D, Ames M, *et al.* 2015. Orchid phylogenomics and multiple drivers of their extraordinary diversification. *Proceedings of the Royal Society B: Biological Sciences* **282**: 20151553.

Grace OM, Buerki S, Symonds MR, *et al.* 2015. Evolutionary history and leaf succulence as explanations for medicinal use in aloes and the global popularity of *Aloe vera*. *BMC Evolutionary Biology* **15**: 29.

Guo J, Xu W, Hu Y, *et al.* 2020. Phylotranscriptomics in Cucurbitaceae reveal multiple whole-genome duplications and key morphological and molecular innovations. *Molecular Plant* **13**: 1117–1133.

Guralnick LJ, Cline A, Smith M, Sage RF. 2008. Evolutionary physiology: the extent of C_4 and CAM photosynthesis in the genera *Anacampseros* and *Grahamia* of the Portulacaceae. *Journal of Experimental Botany* **59:** 1735–1742.

Guralnick LJ, Ting IP, Lord EM. **1986**. Crassulacean acid metabolism in the Gesneriaceae. *American Journal of Botany* **73**: 336–345.

Heyduk K, McAssey EV, Leebens-Mack J. **2022**. Differential timing of gene expression and recruitment in independent origins of CAM in the Agavoideae (Asparagaceae). *New Phytologist* **235**: 2111–2126.

Horn JW, Xi Z, Riina R, *et al.* 2014. Evolutionary bursts in *Euphorbia* (Euphorbiaceae) are linked with photosynthetic pathway: evolutionary bursts in *Euphorbia*. *Evolution* **68**: 3485–3504.

Klak C, Hanáček P, Bruyns PV. **2017**. Out of southern Africa: origin, biogeography and age of the Aizooideae (Aizoaceae). *Molecular Phylogenetics and Evolution* **109**: 203–216.

Li M-H, Liu D-K, Zhang G-Q, *et al.* 2019. A perspective on crassulacean acid metabolism photosynthesis evolution of orchids on different continents: *Dendrobium* as a case study. *Journal of Experimental Botany* **70**: 6611–6619.

Li Z-Z, Lehtonen S, Martins K, Gichira AW, Wu S, *et al.* 2020. Phylogenomics of the aquatic plant genus *Ottelia* (Hydrocharitaceae): implications for historical biogeography. *Molecular Phylogenetics and Evolution* **152**: 196939.

Liede-Schumann S, Grimm GW, Nürk NM, Potts AJ, Meve U, Hartmann HEK. **2020**. Phylogenetic relationships in the southern African genus *Drosanthemum* (Ruschioideae, Aizoaceae). *PeerJ* **8**: e8999.

Liede-Schumann S, Reuss SJ, Meve U, *et al.* 2022. Phylogeny of Marsdenieae (Apocynaceae, Asclepiadoideae) based on chloroplast and nuclear loci, with a conspectus of the genera. *Taxon* **71**: 833–875.

Luján M, Oleas NH, Winter K. **2022**. Evolutionary history of CAM photosynthesis in neotropical *Clusia*: insights from genomics, anatomy, physiology and climate. *Botanical Journal of the Linnean Society* **199**: 538–556.

Massoni J, Couveur TLP, Saquet H. 2015. Five major shifts of diversification through the long evolutionary history of Magnoliidae (angiosperms). *BMC Evolutionary Biology* **15**: 49.

McKain MR, McNeal JR, Kellar PR, Eguiarte LE, Pires JC, Leebens-Mack J. **2016**. Timing of rapid diversification and convergent origins of active pollination within Agavoideae (Asparagaceae). *American Journal of Botany* **103**: 1717–1729.

Messerschmid TFE, Klein JT, Kadereit G, Kadereit JW. 2020. Linnaeus's folly – phylogeny, evolution and classification of *Sedum* (Crassulaceae) and Crassulaceae subfamily Sempervivoideae. *Taxon* **69**: 892–926.

Mok D, Leung A, Searles P, Sage TL, Sage RF. 2023. CAM photosynthesis in *Bulnesia retama* (Zygophyllaceae), a non-succulent desert shrub from South America. *Annals of Botany* (this volume, in review).

Naumann J, Salomo K, Der JP, Wafula EK, Bolin JF, *et al.* 2013. Single-copy nuclear genes place haustorial Hydnoraceae within Piperales and reveal a Cretaceous origin of multiple parasitic angiosperm lineages. *PLoS ONE* **8:** e79204.

Ocampo G, Columbus T. 2010. Molecular genetics of suborder Cactineae (Caryophyllales), including insights into photosynthetic diversification and historical biogeography. *American Journal of Botany* **97:** 1827–1847.

Paton A, Mwanyambo M, Culham A. **2018**. Phylogenetic study of *Plectranthus, Coleus* and allies (Lamiaceae): taxonomy, distribution and medicinal use. *Botanical Journal of the Linnean Society* **188:** 355–376.

Pelser PB, Kennedy AH, Tepe EJ, *et al.* 2010. Patterns and causes of incongruence between plastid and nuclear Senecioneae (Asteraceae) phylogenies. *American Journal of Botany* **97**: 856–873.

Petrova G, Moyankova D, Nishii K, *et al.* 2015. The European paleoendemic *Haberlea rhodopensis* (Gesneriaceae) has an Oligocene origin and a Pleistocene diversification and occurs in a long-persisting refugial area in southeastern Europe. *International Journal of Plant Sciences* **176**: 499–514.

Roalson EH, Roberts WR. **2016**. Distinct processes drive diversification in different clades of Gesneriaceae. *Systematic Biology* **65**: 662–684.

Ruhfel BR, Bove CP, Philbrick CT, Davis CC. 2016. Dispersal largely explains the Gondwanan distribution of the ancient tropical clusioid plant clade. *American Journal of Botany* **103:** 1117–1128.

Silvera K, Santiago LS, Cushman JC, Winter K. 2009. Crassulacean acid metabolism and epiphytism linked to adaptive radiations in the Orchidaceae. *Plant Physiology* **149**: 1838–1847.

Silvera K, Santiago LS, Cushman JC, Winter K. **2010**. The incidence of crassulacean acid metabolism in Orchidaceae derived from carbon isotope ratios: a checklist of the flora of Panama and Costa Rica: CAM in the Orchidaceae. *Botanical Journal of the Linnean Society* **163**: 194–222.

Silvera K, Santiago LS, Winter K. **2005**. Distribution of crassulacean acid metabolism in orchids of Panama: evidence of selection for weak and strong modes. *Functional Plant Biology* **32**: 397–411.

Smith JAC, Winter K. 1996. Taxonomic distribution of crassulacean acid metabolism. In: **Winter K, Smith JAC**, eds. *Crassulacean acid metabolism: biochemistry, ecophysiology and evolution*. Berlin: Springer-Verlag, 427–436.

van der Kerke SJ, Shrestha B, Ruhlman TA, Weng M-A, Jansen RK, *et al.* 2019. Plastome based phylogenetics and younger crown age in *Pelargonium. Molecular Phylogenetics and Evolution* **137**: 33–43.

Wang N, Yang Y, Moore MJ, *et al.* 2019. Evolution of Portulacineae marked by gene tree conflict and gene family expansion associated with adaptation to harsh environments. *Molecular Biology and Evolution* **36**: 112–126.

Wang Y, Zhang C-F, Ochieng Odago W, *et al.* 2023. Evolution of 101 Apocynaceae plastomes and phylogenetic implications. *Molecular Phylogenetics and Evolution* **180**:107688.

Wei X, Qi Y, Zhang X, Luo L, Shang H, Wei R, Liu H, Zhang B. 2017. Phylogeny, historical biogeography and characters evolution of the drought resistant fern *Pyrrosia* Mirabel (Polypodiaceae) inferred from plastid and nuclear markers. *Scientific Reports* **7**:12757.
